# Developing strategies to improve fidelity of delivery of, and engagement with, a complex intervention to improve independence in dementia: a mixed methods study

**DOI:** 10.1186/s12874-020-01006-x

**Published:** 2020-06-12

**Authors:** Holly Walton, Aimee Spector, Anna Roberts, Morgan Williamson, Jem Bhatt, Ildiko Tombor, Susan Michie

**Affiliations:** 1 Department of Applied Health Research, University College London, 1-19 Torrington Place, London, WC1E 7HB UK; 2 Department of Clinical, Educational and Health Psychology, University College London, 1-19 Torrington Place, London, WC1E 7HB UK; 3 Department of Behavioural Science and Health, University College London, 1-19 Torrington Place, London, WC1E 7HB UK; 4 School of Social Sciences, University of Westminster, 115 New Cavendish Street, London, W1W 6UW UK

**Keywords:** Fidelity of delivery, Engagement, Behaviour change, Dementia, Qualitative, Observation, Mixed methods, Implementation, Complex health intervention

## Abstract

**Background:**

It is important to evaluate fidelity of delivery and engagement during feasibility trials. However, there is little guidance on how to systematically develop strategies to improve implementation if problems arise. We aimed to: 1) Assess fidelity of delivery and engagement, 2) Identify factors influencing fidelity of delivery and engagement, and 3) Develop strategies to improve fidelity of delivery of, and engagement with, a complex intervention to improve independence in dementia, within a feasibility trial.

**Methods:**

A mixed methods evaluation of an intervention that aimed to improve independence in dementia. To assess fidelity of delivery and engagement, observation and self-report methods were used: 60% of audio-recorded intervention sessions were transcribed and reliably rated for fidelity. Providers (*n* = 12) and people with dementia/supporters (*n* = 34) were asked to complete checklists after each session. Descriptive statistics were used to analyse the data. To identify factors influencing fidelity and engagement, one-to-one semi-structured interviews were conducted with providers (*n* = 8), people with dementia (*n* = 7) and supporters (*n* = 7). Thematic analysis and content analysis were used to analyse data. To develop strategies, we followed four steps proposed by the authors of the Behaviour Change Wheel (1. Understanding the behaviour, 2. Identifying intervention functions, 3. Specifying intervention content, 4. Identifying mode of delivery).

**Results:**

Researcher ratings indicated moderate fidelity and provider/participant ratings indicated high fidelity of delivery. Knowledge, providers’ attributes, ease of adaptation of the intervention in relation to participants’ needs and logistical considerations influenced fidelity. We developed four strategies to improve fidelity of delivery of PRIDE: 1) showing a video, 2) giving an instruction sheet, 3) giving time to practice and 4) providing continued support. Participants reported high levels of engagement. Participants’ attributes, capability and opportunity influenced engagement. We developed four strategies to improve engagement with PRIDE: 1) a session summary document, 2) clear instructions, 3) time to practice activity and 4) providing regular compulsory telephone support.

**Conclusion:**

Fidelity of delivery and engagement are complex behaviours. This manuscript provides an example of how the Behaviour Change Wheel can be used during a feasibility trial to systematically develop strategies to improve implementation of complex interventions.

## Contributions to the literature

- These findings highlight the complexity of fidelity of delivery and engagement behaviours in complex health interventions.
- This is one of few examples which have used fidelity data to identify issues and develop evidence-based strategies to improve intervention delivery and engagement during a feasibility trial.
- This study provides an example of how mixed methods evaluations can be used to develop strategies to improve implementation for a complex dementia intervention.

## Background

Complex health interventions have the potential to improve outcomes for intervention participants. These complex health interventions are often adapted, e.g. to facilitate the implementation of interventions in a different country [1]. Adaptations may also be considered prior to evaluating an intervention. For example: MRC guidance on developing and evaluating complex interventions states that problems with an intervention (e.g. acceptability, compliance and delivery) can be identified during the feasibility trial and refined or adapted before being delivered and evaluated in an RCT [2]. However, there is little guidance on how to adapt interventions systematically to refine interventions based on problems identified during a feasibility trial.

Findings from evaluations of complex health interventions indicate that interventions are often not delivered as planned (termed ‘fidelity of delivery’) [3–5]. The term engagement is used in this paper as an umbrella term [6] to refer to whether participants understand the information (termed ‘receipt’) and can put their plans into practice (termed ‘enactment’) [7]. Fidelity of delivery refers to the intervention providers’ performance and engagement relates to the participants’ performance. To maximise the potential for effectiveness, it is necessary to understand whether interventions are delivered as planned and engaged with, and develop ways to improve fidelity and engagement if implementation problems arise.

Intervention ‘fidelity’ (including fidelity of delivery and engagement) is one element of process evaluations [8]. Process evaluation is recommended at all stage of intervention evaluation, including feasibility, effectiveness and implementation. Fidelity of delivery and engagement are complex behaviours with many underlying mechanisms. For example, environmental, organisational and individual characteristics have been found to influence fidelity of, and engagement with dementia interventions and interventions in general [9–18]. To understand how an intervention is implemented, the Medical Research Council (MRC) guidance [8], together with previous research [15, 19] propose that mixed methods evaluations of interventions are needed to provide a detailed understanding of intervention functions. Quantitative methods such as observation and self-report can be used to find out whether interventions are delivered as planned (fidelity of delivery) or engaged with [6]; with one method of fidelity of delivery being to audio-record sessions and transcribe and rate a random proportion against a pre-defined intervention checklist [4, 7]. To overcome limitations of individual methods, the use of multiple methods are recommended [20–23]. Alternatively, qualitative methods can be used to gain an in-depth understanding of factors influencing fidelity, including reasons for differences in fidelity or engagement across participants, providers and contexts [8, 23]. By integrating data from both quantitative and qualitative studies, strategies to improve fidelity of delivery of, and engagement with, a feasibility trial could be developed.

Behaviours take place within complex systems [24], therefore changing behaviour is difficult [25]. To develop evidence-based effective strategies for changing behaviour, theory needs to be drawn upon. To take complexities of fidelity and engagement into account, theory needs to include intrapersonal, interpersonal and environmental factors. Individual behaviour change theories are not suitable for this purpose. Instead, a framework of behaviour change can be applied. One such integrated framework is the Behaviour Change Wheel (BCW) [26]. The BCW has three levels: The COM-B (Capability, Opportunity, Motivation – Behaviour) model, nine intervention functions and seven policy categories. The BCW provides a systematic approach to intervention development that can be applied to different populations and behaviours and has previously been used to change behaviours related to fidelity (e.g. [27]) and engagement (e.g. [28–30]). However, to the authors’ knowledge, no research has followed the stages proposed by the authors of the BCW to develop strategies to improve fidelity of delivery of, and engagement with, a feasibility trial.

Previous research has used mixed methods to explore fidelity of delivery of, and engagement with complex health interventions (e.g. [15, 31, 32]). However, to the authors’ knowledge, this is the first study to integrate findings to develop behavioural strategies to improve fidelity of delivery of, and engagement with, a complex dementia intervention. This study extends previous research by using a theory-based, systematic method to develop preliminary strategies which could be used to improve fidelity of delivery of, and engagement with, a future PRIDE RCT.

The strategies outlined in this manuscript are to be considered within the context of improving the Promoting Independence in Dementia (PRIDE) intervention [33, 34]. The PRIDE intervention encourages people living with dementia and supporters (family member/spouse/friend) to identify idiosyncratic goals to increase independence. The PRIDE feasibility trial tested a three session intervention delivered by dementia advice workers (staff working in or alongside memory clinics; here referred to as ‘providers’) to people with dementia and their supporter [33]. The intervention is a manualised, tailored intervention. Dyads (the person living with dementia and their supporter) chose three topics to work on. Topic options included: 1) Keeping mentally active, 2) Keeping physically active, 3) Keeping socially active, 4) Making decisions, 5) Getting the message across, 6) Receiving a diagnosis of dementia and 7) Keeping healthy. In the first session, providers provided information and encouraged participants to choose three topics and plan one or more activities to work on. In the following sessions, providers and participants reviewed plans and engaged in problem solving before developing new plans [34]. The intervention contained both standardised components that were delivered to all participants and tailored components that were dependent on which topics participants chose. Details of intervention components are reported in the PRIDE fidelity checklist development paper [35].

Using PRIDE as a model, this study aimed to demonstrate one way in which strategies can be developed to improve fidelity of delivery of, and engagement with, a feasibility trial. In this paper we use the PRIDE fidelity assessment as an example. The PRIDE fidelity assessment aimed to:

1. Assess fidelity of delivery of, and engagement with the PRIDE intervention
2. Identify factors influencing fidelity of delivery of, and engagement with the PRIDE intervention
3. Develop strategies to improve fidelity of delivery of, and engagement with the PRIDE intervention.

## Methods

### Ethics

Ethical and research governance requirements were followed. Data were transcribed professionally and all transcripts were fully anonymised. Individuals were unidentifiable from data or resulting outputs. Ethical approval was obtained from the NHS East Midlands – Nottingham 1 Research Ethics committee (REC reference number: 16/EM/0044). Data were accessed by authorised study members and stored securely in a central location.

### Design

**Fig. 1:**
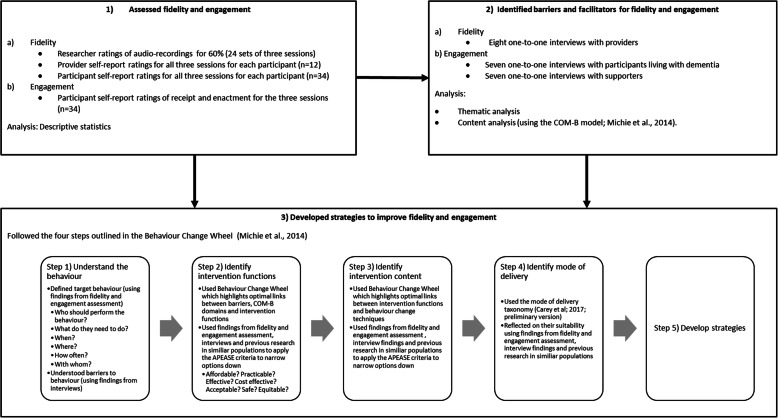
Flow chart outlining how the different parts of the studies link together and how findings from the first two stages informed the third stage

An explanatory sequential mixed methods design was used [23]. Firstly, we conducted a longitudinal observational study assessing how providers delivered the intervention and how participants engaged with the intervention. Then, we conducted a qualitative semi-structured interview study to understand barriers and facilitators to fidelity of delivery and engagement. The findings from both of these studies were then integrated using a behaviour change framework with the purpose of identifying and understanding potential behavioural strategies to improve fidelity of delivery and engagement (see Fig. 1). This is consistent with Creswell’s [23] definition of mixed methods research which involves the collection and integration of quantitative and qualitative data to draw interpretations from the strengths of both datasets to understand research problems.

#### 1) Assessing fidelity of delivery of, and engagement with PRIDE

Further details on this stage of research can be found in the first author’s PhD thesis [36].

##### Measures

To measure fidelity of delivery and engagement, reliable checklists (containing all intervention components) and coding guidelines that can be used by researchers, providers and participants were developed specifically for this study [35].

##### Sample

Sixty percent of all PRIDE intervention sessions: 24 sets (six sets of three sessions from each of the four sites) were randomly sampled. Previous research recommends that researchers monitor fidelity of delivery for 20–40% of intervention sessions [37]. To identify whether fidelity of delivery and engagement varied across participants, providers and sites, data were collected from all four sites. All providers (*n* = 12) and people with dementia/supporters (*n* = 34) were asked to complete fidelity checklists after each session (see [35] for response rates).

##### Procedure

All intervention sessions were recorded (with written consent from people with dementia and supporters). Providers stopped recording if it was inappropriate during the sessions (e.g. participant distress). Audio-recordings were stored securely and transcribed by a professional company. Transcripts were checked for accuracy and fully anonymised (by HW).

HW coded all transcripts to determine whether intervention components were delivered. To identify coder drift (changes in coder agreement over time), a second researcher (MW) double-coded 10% of sets that were not double-coded during measure development (see 35 for details of transcripts double-coded during measure development). Inter-rater agreement was calculated using weighted kappa and percentage agreement. If raters did not achieve weighted kappa scores ≥0.61, further sets were coded and discussed until agreement was reached [35].

Providers were trained to complete fidelity checklists (by HW). This training took place at the end of the PRIDE training day. All providers received the same training for fidelity assessments. Training included details on why it is important to measure fidelity and how to audio-record and complete checklists. Provider and participant checklists were returned by email, via a secure electronic system (providers only) or post (using prepaid envelope).

##### Analysis method

Details on the development of coding guidelines and scores are reported in the PRIDE fidelity checklist development paper (see [35]).

Descriptive statistics were used to assess the percentage of standardised components that were delivered as planned per session (fidelity of delivery). These were compared across sessions (Session 1, Session 2, Session 3), providers and sites. Fidelity of delivery was assessed using data from researchers, providers and the person with dementia (resulting in three fidelity scores for each session). We calculated a percentage and total score for the number of standardised components delivered (80–100% high fidelity, 51–80% moderate fidelity, < 50% low fidelity) [7]. We rated components according to whether they were ‘done’/‘definitely happened’ (Score 2), ‘done to some extent’/‘possibly happened’ (Score 1), ‘not done’/‘didn’t happen’ (Score 0), ‘not applicable’ (Coded 97), ‘missing (Coded -999), or ‘unclear’ (Coded 10). We measured the delivery of tailored topics and components (for Session 1 and Session 2) by calculating the average number of tailored topics delivered in a session and number of tailored components delivered for each topic.

Descriptive statistics were used to assess levels of engagement. We measured intervention receipt (‘information was clear and easy to understand’/‘understanding how to put plans into practice’) and intervention enactment (‘recorded activities on calendar’/‘practiced and used information and skills learnt’). Engagement was assessed using data from the person with dementia’s self-reported checklists. We compared percentages of those who chose ‘yes’ (Score 2), ‘to some extent’ (Score 1), ‘no’ (Score 0), ‘not applicable’ (Coded 97), ‘missing’ (Coded − 999) or ‘unclear’ (Coded 10) across providers and sites.

Missing data, unclear data and components that were not applicable were scored as ‘0’ (not done). This was to ensure that conservative estimates of fidelity of delivery and engagement were provided and to ensure that findings was comparable across providers and sites.

#### 2) Identifying barriers and facilitators to fidelity of delivery of and engagement with PRIDE

Further details on this stage of research can be found in the first author’s PhD thesis [36].

##### Sample

We used purposive opportunity sampling to ensure that providers and people with dementia-supporter dyads were recruited from all four sites. PRIDE providers and participants were invited to take part.

In total, eight providers, seven people with dementia and seven supporters were recruited (see Table 1).

**Table 1:** Demographic characteristics of providers, people with dementia and supporters for one-to-one interviews

Demographic characteristics	Number of providers (total ***N*** = 8)	Number of people with dementia (total ***N*** = 7)	Number of supporters (total N = 7)
Gender			
Female	7	2	5
Male	1	5	2
Experience in years: Mean, SD (range)	9.7, 12.0 (1.5–37)	N/A	N/A
Age: Mean, SD (range)	N/A	79.6, 3.2 (74–82)*	71.7, 15.4 (39–84)
Job roles			
Dementia advisor	4	N/A	N/A
Memory nurse	3	N/A	N/A
Researcher	1	N/A	N/A
Sites**			
Site A	3	2	2
Site B	1	1	1
Site C	1	2	2
Site D	3	2	2

*Missing: n = 2

**To ensure site anonymity, site numbers have been shuffled up so that sites 1-4 do not directly correspond to sites A-D

When potential participants expressed an interest, both members of the dyad were asked if they would like to take part. We interviewed people with dementia and their supporters separately where possible. Both the participant and supporter were present in seven interviews (three person with dementia interviews and four supporter interviews).

##### Measures

Two semi-structured interview guides were developed. The interview schedule for providers was developed to explore barriers and facilitators to the delivery of PRIDE, and experiences of delivery (see Supplementary materials 1). The interview schedule for people with dementia and supporters explored experiences of participating, and barriers and facilitators to engaging with PRIDE (see Supplementary materials 2). Questions mapped onto COM-B [26] and Theoretical Domain Framework (TDF) domains [38]. The PRIDE team and co-authors (AS/IT/SM) provided feedback on questions. We iteratively revised the interview schedules throughout data collection.

##### Procedure

Providers were invited to participate after their last dyads’ final intervention session. People with dementia and their supporters were invited to participate during the follow-up session. Potential participants were contacted by telephone to provide more details and arrange a date and convenient location for the interviews (e.g. participants’ homes or workplace). To ensure that participants were still familiar with the intervention, we aimed to conduct interviews 1–2 weeks after the dyads’ final session, or as soon as possible after the provider delivered their last session. In practice, this was not possible due to timing of follow-up visits. Therefore, interviews took place as soon as possible after the dyads received information about the interview study.

Participants gave written informed consent (including consent for audio-recording) at the beginning of the interview. All participants were in the mild stages of dementia and could provide consent. Supporters also provided secondary consent for the person with dementia, as requested by the ethics committee. We did not collect demographic characteristics unless they were volunteered during the interviews. Participants’ age and providers’ amount of experience were prompted and recorded [39].

Twenty-two interviews were conducted by HW (12–93 min). All interviews were audio-recorded. To prompt discussion, the dyads’ copy of the PRIDE intervention manuals, intervention sheets and fidelity checklists were used. For example, the PRIDE manual goal-setting page was sometimes used to prompt discussion around which activities were chosen. Audio-recordings were transcribed verbatim by a professional company. Transcripts were checked for accuracy and fully anonymised (names and places). Codes were assigned to each participant (e.g. P1: Person with dementia 1).

### Analysis method

To analyse interview data, thematic analysis and content analysis were used.

#### Thematic analysis

This study combined inductive thematic analysis (as proposed by Braun and Clarke) [40] with elements of deductive thematic analysis (e.g. the use of a coding frame) [41]. This approach would fit within a ‘medium Q thematic analysis approach’. [42] The methods of thematic analysis were facilitated by the iterative categorisation method; a systematic, rigorous and transparent technique for sorting data [43].

Transcripts were read and re-read to become familiar with the data. Line-by-line coding was then conducted for 12 interviews (four provider interviews, four participant interviews and four supporter interviews) to generate initial codes. We then developed an initial coding framework from this inductive coding (one for delivery, one for engagement) [41]. Two researchers (HW/JB) independently applied the coding framework to three transcripts (one provider, one person with dementia, one supporter). Differences were discussed and resolved. Minor changes were made to the coding framework. Then, HW coded all transcripts (*n* = 22) using the final coding framework using NVivo 11. From this coding, themes were developed, reviewed, defined and labelled. A table of themes, sub-themes and example quotes was created for both fidelity of delivery and engagement. Themes and example quotes were reviewed by JB and discussed with the wider research team.

Previous research has suggested that reliability assessments may not be appropriate [40, 44] as they may restrict coding flexibility and the identification of new findings [45]. Instead, to enhance trustworthiness of data analysis and analysis interpretation, a second researcher (JB) was involved in applying the coding frame and peer reviewing coding and summaries [46–49].

#### Content analysis

Deductive content analysis (categorisation of data to pre-defined categories) [50] was also used to identify which COM-B domains were frequently reported as barriers/facilitators to delivery and engagement. Data was categorised into: capability (psychological or physical), opportunity (physical or social) and motivation (automatic or reflective) [26]. All of the raw data quotes were extracted into a spreadsheet and duplicates were removed. Two authors (HW/AR) coded all extracts into COM-B categories and as barriers/facilitators. Discrepancies were resolved through discussion. Quotes that did not fit into any domains were coded as ‘none’. More than one domain could be coded for each quote. Frequency of each category was identified.

### 3) Developing strategies to improve fidelity of delivery and engagement

To improve fidelity of delivery of, and engagement with, PRIDE, we developed initial strategies using the steps proposed by the authors of the BCW [25]. These strategies were designed to inform improvements to a future PRIDE RCT. Steps include: 1) Understanding the behaviour, 2) Identifying intervention functions and policy categories, 3) Specifying intervention content in terms of Behaviour Change Techniques (BCTs: active intervention ingredients) and 4) Identifying a mode of delivery [25].

Firstly, the target behaviours were defined and barriers and facilitators for these behaviours were identified (Step 1). To identify appropriate intervention functions, the BCW was used to highlight optimal links between barriers/facilitators and intervention functions (Step 2). To identify appropriate behaviour change techniques, the BCW was used to highlight optimal links between intervention functions and behaviour change techniques (Step 3). Suitable modes of delivery for individual strategies were then identified (Step 4) and strategies to improve fidelity of delivery and engagement were developed (Step 5) [25]. Findings from the quantitative fidelity/engagement assessment (stage 1) and qualitative findings (stage 2), together with previous research in similar populations were used to identify and narrow down appropriate solutions by judging the suitability of different options (in Steps 2–4). Each of these steps (including how findings from both the quantitative and qualitative studies were integrated) are described in more detail in Fig. 1. Further details on this stage of research can be found in the first author’s PhD thesis [36].

## Results

### 1) Assessing fidelity of delivery of, and engagement with PRIDE

#### Fidelity of delivery

##### Standardised components

Ratings from transcripts of audio-recordings indicated that a mean of 69% of components were delivered in Session 1 (range: 13.6–86.4%), 57.7% in Session 2 (range: 41.7–83.3%) and 54.9% in Session 3 (range: 25–95.8%). Provider self-report ratings indicated that a mean of 85% of components were delivered in Session 1 (range: 22.7–100%), 84.3% in Session 2 (range: 61.1–100%) and 86.5% in Session 3 (range: 62.5–100%). Participant self-report ratings indicated that a mean of 89.8% of components were delivered in Session 1 (range: 59.1–100%), 90.1% in Session 2 (range: 50–100%), and 92.5% in Session 3 (range: 50–100%). Findings indicate that providers delivered PRIDE with moderate to high fidelity; with researcher ratings indicating the lowest levels and participant self-report ratings indicating the highest levels of fidelity. Mean fidelity for each session varied across sites and providers across all three sources of rating (see Supplementary materials 3 or [36] for further details).

##### Tailored components

Ratings from transcripts of audio-recordings indicated that a mean of 1.9 topics and 4.6 components were delivered in Session 1 and 1.1 topics and 3.0 components in Session 2. Provider self-report ratings indicated that a mean of 2.4 topics and 7.9 components were delivered in Session 1 and 2.2 topics and 7.8 components in Session 2. The number of tailored topics and components delivered varied across sites and providers (see Supplementary materials 4 or [36] for further details).

#### Engagement

Findings from the self-report checklists completed by participants with dementia indicated that the mean level of receipt was 85.9% for Session 1 (range: 0–100%), 87.5% for Session 2 (range: 50–100%) and 90.6% for Session 3 (range: 50–100%). The mean level of enactment was 81.3% for Session 2 (range: 0–100%) and 82.8% for Session 3 (range: 0–100%). Findings indicate that participants understood information and were able to put plans into practice (see Supplementary materials 5 or [36] for further details).

### 2) Identifying barriers and facilitators to fidelity of delivery of and engagement with PRIDE

#### Fidelity of delivery

##### Thematic analysis

Four themes were developed. Examples quotes are shown in Table 2. More details from this analysis can be found in the first author’s PhD thesis [36].

I. Providers’ knowledge about how to deliver PRIDE

**Table 2:** Themes, sub-themes and example quotes for fidelity of delivery

Theme	Sub-theme	Example quote
Fidelity of delivery		
I. Providers’ knowledge	1) Prior knowledge	*“Well I can’t really say for the others but we’re all really experienced […] A bit more confident and thinking I can do this.”* (DAW 3, Site A)
	2) Skills to deliver PRIDE	*“I think from the training point of view […] I came away thinking I can fill those forms in now but I can’t deliver that. So it may need to be more about the delivery, the actual how you want it delivering, the key points, and this is so much information and maybe it would be useful for like bullet points, key points, to be pulled out a bit.”* (DAW 2, Site A)
II. Providers’ personal attributes	3) Beliefs about PRIDE as part of job	*“It’s something that we are actually doing. We do encourage people to do things that they maybe didn’t do before and to look at what they enjoy doing or they used to enjoy previously and try and engage with that so once you realise that it makes it a lot less daunting.”* (DAW 1, Site A)
	4) Personal characteristics	*“I think that’s just my personality. Because I kind of felt if I don’t do it that way then I’m going to miss something and I was a little bit conscious of the checklist that we had to do afterwards, thinking I want a tick in every one of the boxes. Again that’s my personality.”* (DAW 1, Site A)
	5) Feelings about delivery	*“I was quite nervous because of the recording, I was quite nervous just in case I was missing something that was important. I think after the first session I felt a bit better. Like okay, we’ve actually set a goal, we’ve done all this, I think I talk about herself, what she likes, so that’s fine.”* (DAW 6, Site D)
III. Adaptation of PRIDE in relation to participants’ needs	6) Ease of adaptation with fidelity	*“It was trying […] and thinking right, there is no right and then there is no wrong […] it’s about people’s choice, what is stated in a manual, when you’re working with dementia, cannot always be followed […] So, we can only deliver it how we feel is best for that person when we arrive […] That’s my opinion anyway.”* (DAW 4, Site D)
	7) Participant engagement	*“Well my service users did [help delivery] because they were quite keen. They definitely helped with the momentum without having to try and give too much encouragement and go and see them and things had happened […] So that helped.”* (DAW 1, Site A)
IV. Logistical considerations	8) Organisational constraints	*“I mean it’s something I’d like to do but, like I said, it’s quite difficult when you’ve got other job commitments. I found it a lot easier once I started working part time […] when I was with the [Organisation 1] it was full time plus extra hours […] So I’d be working till half four then going to do an intervention, getting home at half six seven with the traffic. That was really draining. My new job, because I was working part time there was a lot more flexibility. And even just delivering intervention during the day like the traffic made a massive difference”* (DAW 8, Site B)
	9) Social support for delivery	*“We’ve used each other’s experience of, you know, what’s worked and what hasn’t. […] I think to begin with, because [Name of DAW] was the first one who did it, we were all like listening to how she’d delivered it and that was really useful and helpful. So I think between us we’ve sort of taken little bits of each other’s experience.”* (DAW 2, Site A)

Providers’ previous qualifications and/or experience of working with people with dementia helped them to deliver PRIDE with fidelity by increasing confidence and ability to tailor the intervention. Prior knowledge of individual participants and the local environment helped them to deliver PRIDE as planned (e.g. knowledge of activities in local area).

Whilst providers understood how to complete forms, a lack of knowledge limited their skills to deliver PRIDE or use the manual; thus making it more difficult to deliver the intervention with fidelity. Providers expressed a need for further training on the practical elements of delivery (e.g. delivering key information and adapting this to participants, or delivering PRIDE using the manual). Practice and familiarisation with the manual and practice developed confidence in delivering PRIDE, with delivery becoming easier over time.

II. Providers’ personal attributes

Providers’ perceptions that PRIDE was different to their job role made it more difficult for providers to deliver PRIDE as planned (e.g. PRIDE not being in their remit and not being used to following a manual), whereas similarities made it easier to deliver (e.g. delivering similar content or dealing with similar issues that exist within providers’ current job roles).

Personal characteristics (e.g. feeling conscious about delivering the intervention as planned, wanting to stay longer with participants), and personal views on benefits for themselves (e.g. wanting to learn more, enjoying spending time with people with dementia) and the participants (e.g. having potential to change post-diagnostic support) influenced delivery.

Some providers were anxious about delivering PRIDE for many reasons including being judged by participants and being recorded/filling in fidelity checklists. On the other hand, perceived self-confidence facilitated delivery; including age, work-related experience and experience delivering PRIDE.

III. Adaptation of PRIDE in relation to participants’ needs

There was conflict between trying to deliver PRIDE as planned and delivering PRIDE in the most appropriate way for participants. As it was difficult to deliver PRIDE as planned, providers delivered the intervention flexibly, with participants’ needs in mind. Delivering PRIDE with strict fidelity felt restricting and providers were more comfortable delivering PRIDE using their own style. Having experience helped providers to deliver PRIDE and facilitated the adaptation of PRIDE in relation to participants’ needs, but may also have hindered the delivery of PRIDE as planned in the manual. Providers initially delivered the intervention with strict fidelity but became less reliant on the manual and delivered PRIDE flexibly over time.

Participants’ understanding, motivation and engagement influenced fidelity of delivery. There was a lack of consensus among providers regarding the appropriateness of PRIDE for people with different stages of dementia. PRIDE was perceived as suitable to those with a diagnosis of mild dementia if the characteristics of dementia (e.g. level of cognitive impairment) were considered when delivering the intervention. PRIDE was seen as more difficult to deliver in some situations (e.g. supporters not being present in sessions).

IV. Logistical considerations

PRIDE mostly fitted well around providers’ work commitments. Facilitators of fidelity included having a supportive work environment, managing their own diaries, and the allocation of appropriate time for each dyad. Lack of time to prepare for sessions and travel to dyads, and difficulties scheduling three sessions at the right time in the month hindered fidelity of delivery.

The PRIDE research team, site researchers and other providers facilitated fidelity of delivery. Peer support from other providers offered an opportunity to share experiences, gain knowledge about delivering PRIDE and reduce anxieties. More support with technical difficulties from researchers or peers may have been beneficial (e.g. support with Dictaphones). There was no consensus regarding what role supporters should have; with findings indicating that the presence of a supporter should depend on individual needs. Providers felt that supporters facilitated delivery (e.g. providing practical support for engaging in activities). Others reported that sometimes supporters were involved too much (e.g. telling own story in session which reduced intervention time) or too little (e.g. not being there during sessions made it hard to make progress).

##### Content analysis

For fidelity, frequent barriers related to social opportunity (*n* = 131), physical opportunity (*n* = 123) and psychological capability (*n* = 85). Frequent facilitators related to social opportunity (*n* = 162), physical opportunity (*n* = 110), reflective motivation (*n* = 107) and psychological capability. The frequency of occurrences of COM-B domains are reported in Table 3 (see Supplementary materials 6/7).

**Table 3:** Frequency of occurrences of COM-B domains in total and by barriers to and facilitators for fidelity of delivery

COM-B	Number of occurrences of domains within quotes		
Component and domain	Total	Barriers	Facilitators
Capability			
Psychological	139	85	85
Physical	1	1	0
Opportunity			
Physical	188	123	110
Social	214	131	162
Motivation			
Automatic	74	38	45
Reflective	119	28	107
None	45	–	–

Note: Barriers and facilitators do not add up to the total due to some quotes being coded both as barriers and facilitators. More than one domain could be coded for each quote

#### Engagement

##### Thematic analysis

Three themes were developed. Examples quotes are shown in Table 4. More details from this analysis can be found in the first author’s PhD thesis [36].

I. Participants’ attributes

**Table 4:** Themes, sub-themes and example quotes for engagement

Theme	Sub-theme	Example quote
Engagement		
I. Participants’ attributes	1) Preferences for PRIDE activities	*“Yes, it was very good, but he is so thorough. He was in his working life. Do you know in his…? He couldn’t just say, went to so-and-so […] It’s got to be everything he done that day. He’s a bit obsessive […] I wouldn’t say obsessive […] so to him, there wasn’t enough space.”* (S1, Female)
	2) Beliefs about PRIDE	*“I thought it was just what I required […] it was encouragement to do what we’ve just been talking about, being involved with people […] I can’t think of anything that’s more important, no.”* (P3, Male)
	3) Feelings about PRIDE	*“Well, I wouldn’t go out on my own because I’m frightened of falling and things”* (P6, Female)
II. Participants’ capability	4) Physical health	*“I was always interested in playing bowls, I don’t think I could do it now because I’ve got a dodgy knee, but I could go and watch.”* (P1, Male)
	5) Cognitive factors	*“Well the other thing that gets in my way is I’m not good at initiating things […] Is the thing. If I don’t know what I’m doing I just don’t do it”* (P2, Male)
III. Participants’ opportunity to engage	6) Accessibility	*“If I could find an easy, accessible bridge club that would be good.”* (P2, Male)
	7) Social support	*“[DAW] put the thing in my mind but [Name of supporter], sort of, looked out for different groups like that […] And seeing whether we can get in there.”* (P1, Male)
	8) Activity characteristics	*“We looked into group, walking groups, but the one that they do, it’s not just dementia they deal with at [Place 4], it’s all elderly people over 60, I think. They have walking groups, but they’re 3–4 mi. Well, that’s too much for us”* (S1, Female)

People with dementia and supporters reported taking part in activities that they wanted or like to do (e.g. those that they have experience with, those that are convenient and easy, or social activities) and avoided activities they disliked. However, if participants were unable to perform the activities, liking activities was not sufficient to influence engagement. Personality traits (including competitiveness, sportiness, sociability and thoroughness) influenced decisions to engage in activities.

People with dementia and supporters’ beliefs about PRIDE facilitated engagement. They felt that PRIDE and its components were helpful in encouraging them to do activities, having someone to talk to and for helping others. If participants felt that PRIDE was relevant to them, this facilitated engagement. Despite the manual facilitating engagement, participants reported that some aspects of the manual were not relevant for them. Some participants felt that the manual/intervention content would become more relevant as symptoms progress.

Enjoyment of PRIDE sessions and spending time with the provider, and enjoyment of taking part in their chosen activities helped participants to engage. However, anxiety towards activities chosen during PRIDE sessions was perceived to limit engagement activities for people with dementia and their supporters. Conversely, anxieties (e.g. about doing activities, travelling to activities and using technology) and negative feelings (in relation to memory impairment, inability to go out and do things and not enjoying company) limited engagement.

II. Participants’ capability

Physical health problems challenged engagement with certain activities whereas good health facilitated engagement. Cognitive factors including memory (e.g. not remembering sessions and intervention materials, or memory affecting ability to engage with activities), knowledge (e.g. understanding information in sessions and not knowing how to get to activities, or do activities) and communication skills were perceived to influence engagement. Familiarity with activities was perceived to facilitate engagement. Participants discussed engaging in activities that they do not do as frequently as they used to, but that they still have the capability to do.

III. Participants’ opportunity to engage

Accessibility, including accessibility of PRIDE materials and activities influenced engagement. Participants felt that PRIDE paperwork was easy to complete and the manual was easy to understand; thus facilitating engagement. The location and accessibility of activities helped or hindered people with dementia and supporters from engaging with activities (e.g. it helped if participants could travel to the activity easily). Having the physical resources needed to engage with activities helped people with dementia and their supporters to do their activities (e.g. using reminders, writing notes, or having the necessary tools). However, cost, time and challenges finding the appropriate equipment hindered engagement.

Practical support from the provider (e.g. encouraging them to do their activity, or giving information) helped participants to engage with the intervention sessions and activities. Practical support from supporters (e.g. setting up activities, researching and organising activities, helping them get to the activity and help to complete forms) also facilitated engagement for people with dementia. Practical support from family members included accompanying the person with dementia to their activity to overcome travel barriers. Emotional support from providers, family members and organisations facilitated engagement. For example, having a positive relationship with their provider facilitated engagement by giving participants someone to talk to during PRIDE. In some cases, further support was perceived to be necessary (e.g. having someone to do the activity with).

##### Content analysis

For engagement, frequent barriers related to physical opportunity (*n* = 102) and psychological capability (*n* = 100). Frequent facilitators related to social opportunity (*n* = 188), reflective motivation (*n* = 144), physical opportunity (*n* = 111) and automatic motivation (*n* = 93). The frequency of occurrences of COM-B domains are reported in Table 5 (see Supplementary materials 8/9).

**Table 5:** Frequency of occurrences of COM-B domains in total and by barriers to, and facilitators for engagement

COM-B	Number of occurrences of domains within quotes		
Component and domain	Total	Barriers	Facilitators
Capability			
Psychological	119	100	30
Physical	35	26	15
Opportunity			
Physical	176	102	111
Social	207	40	188
Motivation			
Automatic	124	50	93
Reflective	167	36	144
None	57		

Note: Barriers and facilitators do not add up to the total due to some quotes being coded both as barriers and facilitators. More than one domain could be coded for each quote

### 3) Developing strategies to improve fidelity of delivery and engagement with PRIDE

#### Fidelity of delivery

Findings from the fidelity assessment and interviews were integrated to develop improvement strategies. Further details can be found in the first author’s PhD thesis [36].

The target behaviour that strategies aimed to improve was that providers would deliver PRIDE components that were infrequently delivered within the PRIDE feasibility trial. These components related to tailoring the intervention to participants (providing resources for chosen topics and discussing these in relation to the participants) and problem solving. Three intervention functions were identified as potentially relevant to include within recommendations to improve key barriers, including: psychological capability (e.g. skills to deliver PRIDE as planned and remembering information from training), social opportunity (e.g. participant engagement and support from researchers) and physical opportunity (e.g. having the appropriate PRIDE resources and time to practice delivering PRIDE) to deliver PRIDE as planned. These were ‘Training’, ‘Modelling’ and ‘Enablement’. One policy category was identified as relevant: ‘Service provision’. Four BCTs from the BCTTv1 [51] were identified to include within recommendations. These were: ‘Social support (unspecified)’ (BCT 3.1), ‘Instruction on how to perform the behaviour’ (BCT 4.1), ‘Demonstration of behaviour’ (BCT 6.1), and ‘Behavioural practice and rehearsal’ (BCT 8.1). Four different types of mode were identified as suitable to deliver the four BCTs. These were: human interactions (e.g. face-to-face or over the telephone), printed materials and digital delivery (see Table 6 for details of each step and rationale).

**Table 6:** Mapping of fidelity findings and previous research onto Steps 1–4 of the BCW [25], along with resulting recommendations

Behaviour Change Wheel step	Summary of outcome	Details of outcome and rationale
1) Understand the behaviour	One target behaviour developed	- **Who?** Providers
		- **What do they need to do?** Deliver components that were infrequently delivered within the PRIDE feasibility trial, including tailoring (providing resources for chosen topics and discussing) and problem solving
		- **When?** During each of the three sessions
		- **Where?** Participants’ home
		- **How often?** To all participants
		- **With whom?** Person with dementia and supporter
	Three COM-B domains were identified as frequent barriers: - Psychological capability - Physical opportunity - Social opportunity	- **Psychological capability** (e.g. skills to deliver PRIDE as planned and remembering information from training)
		- **Physical opportunity** (e.g. appropriate PRIDE resources and time to practice delivering)
		- **Social opportunity** (e.g. participant engagement and support from researchers)
2) Identify intervention functions and policy categories	Three intervention functions were identified: - Training - Modelling - Enablement	• **Training (to improve skills)**
		- Review of 152 education and training interventions for staff involved in dementia care suggests training increases knowledge, staff confidence and facilitates behaviour change [52]
		- Poor training = one reason why interventions not effective [7]
		- Requires more time and money [53]
		- Acceptable as providers spoke about wanting more training in the interviews
		• **Modelling (to show providers how to deliver PRIDE)**
		- Seeing procedures facilitates acquisition of clinical skills [54]
		- Role modelling - acceptable to providers who spoke about wanting more guidance about how to deliver PRIDE in interviews
		• **Enablement (to increase capability or opportunity)**
		- Findings indicated fidelity differed across providers and sites - Interview findings highlighted differences in work environments and social support
		- Development of effective training for behaviour change may include expert clinical supervision/staff champions [52]
		- Broader work environment needs to be facilitative to deliver high-quality person-centred dementia care [55]
		- Acceptable to providers who spoke about importance of social support during interviews - individual training/supervision may be beneficial
	One policy category was relevant	Service provision
3) Identify intervention content (BCTs)	Four BCTs were identified: - Social support (unspecified) (BCT 3.1) - Instruction on how to perform the behaviour (BCT 4.1) - Demonstration of behaviour (BCT 6.1) - Behavioural practice and rehearsal (BCT 8.1)	• **Social support (unspecified) (BCT 3.1)**
		- Interview findings indicated social support from researchers and peers was acceptable
		- Social support was identified as a key theme facilitating fidelity in the interviews
		• **Instruction on how to perform the behaviour (BCT 4.1)**
		- Interventions containing this BCT may improve GPs’ delivery of two recommendations from clinical practice guidelines for back pain management in primary care [56, 57]
		- Interview findings indicated instructions would be acceptable to providers who reported needing more step-by-step guidance on how to deliver practical elements (e.g. adapting PRIDE to participants)
		• **Demonstration of behaviour (BCT 6.1)**
		o Interventions containing demonstration may improve delivery of healthcare interventions [52, 56, 57]
		o Review of 152 dementia education and training interventions found that training interventions which consisted of active learning approaches and examples showing how to deliver an intervention through written materials, video or DVD were useful to demonstrate good practice to staff working with dementia [52]
		o Interview findings indicated that providers wanted more step-by-step guidance on how to deliver PRIDE as planned
		• **Behavioural practice and rehearsal (BCT 8.1)**
		- Interventions using this BCT found to improve delivery of guidelines in primary care (56,557)
		- Providers wanted more opportunities to practice delivering PRIDE components
4) Mode of delivery	Four types of mode were identified as suitable to deliver the four BCTs: - Human interactions (face-to-face) - Human interactions (remote) - Printed materials - Digital delivery	• **Social support (unspecified) (BCT 3.1)**
		- Could be delivered either face-to-face during PRIDE training day or via telephone
		- Providers are based at different sites, so face-to-face contact not always possible - telephone calls maybe more suitable for PRIDE
		• **Instruction on how to perform the behaviour (BCT 4.1)**
		- Could be delivered through human contact, printed materials or digitally - Printed materials may be more suitable in PRIDE as providers spoke about difficulties remembering information
		- Provided on the training day during the interviews
		• **Demonstration of behaviour (BCT 6.1)** - Could be delivered through human contact or digitally
		- All providers need to receive standardized training [58] - Therefore, demonstration could be delivered digitally or by somebody who has been trained to demonstrate the intervention consistently
		• **Behavioural practice and rehearsal (BCT 8.1)** - Could be delivered face-to-face during the PRIDE training day
5) Resulting recommendations	Four recommendations were developed: 1) Show a video of how to deliver PRIDE 2) Give an instruction sheet about how to deliver PRIDE 3) Give providers time to practice delivering PRIDE 4) Provide continued support from researchers for delivery	**1) • Show a video of how to deliver** PRIDE
		- Aims to increase skills, reduce anxieties and improve social support
		- Targets training & modelling using BCT 6.1 - Implemented using a digital mode of delivery (providers shown a video during PRIDE training)
		**2) • Give an instruction sheet about how to deliver PRIDE**
		- Aims to increase providers’ skills and reduce anxieties
		- Targets training, using BCT 4.1
		- Providers would be given a printed instruction sheet summarizing information in manual - clear and step by step for standardized and tailored components
		**3) • Give providers time to practice delivering PRIDE**
		- Aims to increase skills
		- Targets training using BCT 8.1
		- Delivered face-to-face during training (paired up and asked to practice delivering and tailoring based on a case study)
		**4) • Provide continued support from researchers for delivery**
		- Aims to improve social support
		- Targets enablement using BCT 3.1
		- Delivered over the phone + additional phone calls to address individual differences

In total, four strategies were developed to improve fidelity of delivery. These were: 1) show a video of how to deliver PRIDE, 2) give an instruction sheet about how to deliver PRIDE, 3) give providers time to practice delivering PRIDE within the training session, and 4) provide continued support from researchers for delivery. Table 6 (Step 5) provides more details of recommendations and potential implementation.

#### Engagement

The target behaviour was that people with dementia and supporters would carry out the activities that they planned in the first or second PRIDE sessions (e.g. attending an activity group or completing a jigsaw puzzle). Three intervention functions were identified as potentially relevant to include within strategies to improve key barriers, including: physical opportunity (money and time to do the activity, accessible locations and resources to prompt activities) and psychological capability (knowledge and skills about how to do activities and how to organise and carry out activities). These were ‘Education’, ‘Training’ and ‘Enablement’. One policy category was identified as relevant: ‘Service provision’. Five BCTs were identified to include within recommendations. These were: ‘Social support (unspecified)’ (BCT 3.1), ‘Social support (practical)’ (BCT 3.2), ‘Instruction on how to perform the behaviour’ (BCT 4.1), ‘Prompts and cues’ (BCT 7.1), and ‘Behavioural practice and rehearsal’ (BCT 8.1). Three different types of mode of delivery were identified as suitable to deliver the five BCTs. These were: face-to-face human interactions, human interactions remotely via telephone and printed materials (see Table 7 for details of each step and rationale).

**Table 7:** Mapping of engagement findings and previous research to steps 1–4 of BCW [25], and resulting recommendations

Behaviour Change Wheel step	Summary of outcome	Details of outcome and rationale
1) Understand the behaviour	One target behaviour developed	- **Who?** People with dementia and supporters
		- **What do they need to do?** Carry out the activities that they planned in the first or second PRIDE sessions (e.g. attending a group or completing a jigsaw)
		- **When?** In the four weeks between each of the three sessions
		- **Where?** Participants’ home or in community • **With whom?** The provider, supporter and other people
	Two COM-B domains were identified as frequent barriers: - Physical opportunity - Psychological capability	- **Physical opportunity** (e.g. money and time to do the activity, accessible locations and resources to prompt activities)
		- **Psychological capability** (e.g. knowledge and skills about how to do the activities and how to organise and carry out activities)
2) Identify intervention functions and policy categories	Three intervention functions were identified: - Education - Training - Enablement	• **Education (to support engagement with PRIDE)**
		- Participants and supporters in the interviews reported not always knowing or remembering what activities they had chosen to work on during the session
		• **Training (to support engagement with PRIDE)**
		- Training interventions may be acceptable, effective and safe for people with dementia [59]
		- Findings showed that participants reported not always knowing what to do to put their plans into practice
		- PRIDE is already lengthy (three one hour sessions)- so would need to be easily implemented
		• **Enablement (to support engagement with PRIDE)**
		- Previous research suggests enablement empowers people with dementia to make decisions and encourages them to have a go at activities [60]
		- People with dementia and supporters spoke about importance of social support provided by provider, supporters and other people
	One policy category was relevant	Service provision
3) Identify intervention content (BCTs)	Five BCTs were identified: - Social support (unspecified) (BCT 3.1) - Social support (practical) (BCT 3.2) - Instruction on how to perform the behaviour (BCT 4.1) - Prompts and cues (BCT 7.1) - Behavioural practice and rehearsal (BCT 8.1)	• **Social support (unspecified) (BCT 3.1)**
		- Research suggests social support (unspecified) contributed towards an improvement in physical activity for people with dementia [61]
		- Interview findings suggest that social support from the provider facilitated engagement with PRIDE
		• **Social support (practical) (BCT 3.2)** - Research suggests subtle practical support (E.g. helping the person form strategies to do their activities) helps maintain independence and make decisions [60]
		- Interview findings highlighted practical support from many different people facilitated engagement with PRIDE
		• **Instruction on how to perform the behaviour (BCT 4.1)**
		- Research suggests that exercise classes, which include instructions, facilitate engagement with physical activity for people living in residential homes [62]
		- Interview findings indicated that a lack of knowledge about how to do activities made it difficult for some participants to put their plans into practice
		• **Prompts and cues (BCT 7.1)** - Research suggests that prompting the person with dementia improves engagement with interventions or activities [63–68]
		- Prompts would be acceptable as participants spoke about importance of reminders and recommended using sticky notes to highlight relevant sections of the manual or provide summaries between sessions
		• **Behavioural practice and rehearsal (BCT 8.1)**
		- Previous research indicated that exercise classes, which include practice facilitated engagement with physical activity for people with dementia living in residential homes [62]
		- Participants spoke about wanting to engage in activities they were familiar with
4) Mode of delivery	Three types of mode were identified as suitable to deliver the four BCTs: - Human interactions (face-to-face) - Human interactions (remote) - Printed materials	- The BCTs: **Social support (practical)** **(BCT 3.2)** and **Behavioural practice and rehearsal(BCT 8.1)** could be delivered face-to-face by the provider during the PRIDE sessions
		- **Social support (unspecified)(BCT 3.1)** could be delivered over the phone between sessions
		- The BCTs: **Instruction on how to perform the behaviour** **(BCT 4.1)**, and **Prompts and cues** **(BCT 7.1)** could be delivered through the provision of printed materials
		- Findings from the interviews indicated that this would be acceptable to people with dementia and supporters
		- Participants reported that a summary sheet may be helpful, during the interviews.
		- The delivery of these BCTs would require minimal additional resources.
5) Resulting recommendations	Four recommendations were developed: 1) Give participants a session summary document 2) Give participants clear instructions detailing how to do their chosen activities 3) Ensure that there is time within the PRIDE session to practice the chosen activity where possible 4) Provide regular compulsory telephone support from provider	**1) • Give participants a session summary document**
		- Aims to prompt enactment and increase understanding
		- Targets education using BCT 7.1 - Delivered through printed materials - summary document provided to the participants after each session - can be placed on fridge door/in homes somewhere visible. Facilitates involvement of supporters
		**2) • Give participants clear instructions detailing how to do their chosen activities**
		- Aims to prompt enactment, and increase understanding and develop skills
		- Targets training using BCT 4.1
		- Delivered through printed materials - clear step-by-step instructions would be given at the end of each session. Instructions would be created by providers in the session and would require additional time
		**3) • Ensure that there is time within the PRIDE session to practice the chosen activity where possible**
		- Aims to increase skills and accessibility of activities
		- Targets training and enablement using BCTs 8.1 and 3.2
		- Delivered face-to-face - participants could practice in session with support from provider or where not feasible the provider could arrange for the supporter to help the person the first time they do it
		**4) • Provide regular compulsory telephone support from provider** - Aims to prompt enactment and increase understanding - Targets enablement using BCT 3.1
		- Delivered over the phone between sessions to remind of activity and answer any questions they have

In total, four strategies were developed to improve engagement: 1) give participants a session summary document, 2) give participants clear instructions detailing how to do their chosen activity, 3) ensure that there is time within the PRIDE sessions to practice the chosen activity where possible, and 4) provide regular compulsory telephone support from the provider. Table 7 (Step 5) provides more details of recommendations and potential implementation.

## Discussion

### Key findings

This manuscript aimed to outline a mixed-methods process that can be used to systematically develop recommendations to improve fidelity of delivery and engagement. This manuscript outlined fidelity of delivery and engagement findings from an intervention which aimed to improve independence in dementia [33, 34]. Researcher ratings indicated moderate fidelity of delivery and provider and participant rating indicated high fidelity of delivery. Several factors influenced fidelity of delivery including knowledge, personal attributes, skills and a supportive work environment. Engagement among people with dementia and supporters was high. Many factors influenced engagement including personal attributes, capability, and opportunity to engage with PRIDE and related activities. Four strategies to improve fidelity and four strategies to improve engagement were developed using the BCW [25]. Whilst these strategies were developed specifically for PRIDE, the methods used to develop strategies can be applied to other complex interventions (see [36] for further details).

### How findings relate to previous research

This study extends previous knowledge [2] by using a theory-based, systematic method to develop preliminary strategies that could be used to improve fidelity of delivery of, and engagement with, a future PRIDE randomised control trial, as well as other evaluations of complex interventions. The resulting outcomes provide detailed and transparent information about (i) strategies that were developed, and (ii) the associated BCTs and intervention functions [69]. This information could inform PRIDE researchers’ decisions when considering intervention refinement. In addition, the methods outlined in this manuscript can be used more widely by other intervention developers and evaluators to identify problems with implementation during the feasibility stage and to develop strategies to potentially improve fidelity and engagement.

Whilst strategies developed using the Behaviour Change Wheel largely focus on overcoming individual barriers for providers and participants (e.g. knowledge), some strategies also target organisational factors (e.g. providers (not having time within the work role to practice delivering PRIDE as planned). Targeting organisational barriers is important given that these findings, along with previous research findings demonstrate organisational barriers to fidelity of delivery [12, 17, 55] and environmental barriers to engagement [11, 18, 70, 71]. Additionally, the level of detail and adaptation with which strategies need to be implemented will differ for individual participants and providers depending on their individual needs.

Our findings demonstrate the complexity of fidelity of delivery and engagement behaviours. There were many factors found to influence fidelity of, and engagement with, PRIDE; despite PRIDE being moderately delivered as planned and engaged with by participants. Our finding that PRIDE was delivered with at least moderate fidelity supported previous research, which indicates that interventions are often not delivered completely as planned [3–5, 10]. Similarly, our findings of self-reported high levels of engagement refute findings that suggest that older adults find it difficult to engage with information given by healthcare professionals in health appointments [72–74]. Our qualitative findings provided support for research which has suggested that a range of factors including intervention factors, provider factors, participant factors and organisational factors influence both fidelity and engagement [13, 14, 17].

### Strengths and limitations

A key strength of this research is that it used mixed methods. By conducting a thorough interview study alongside the measurement of fidelity and engagement, these findings provided better insights into what was delivered and engaged with and why.

One limitation of the framework used in this research is that links between intervention functions and BCTs are based on expert consensus [25], and some of the identified BCTs may not be effective in particular populations, behaviours or settings. However, as demonstrated, there is some evidence from previous research which took place in different populations, settings and/or behaviours which suggests that the selected intervention functions and BCTs may be effective for improving fidelity of, and engagement with, PRIDE.

### Implications

#### Wider implications

The BCW provided a systematic approach for developing strategies. This behavioural science approach may be appropriate for use in other process evaluations of complex interventions. The mixed-methods process outlined in this manuscript can be applied by other intervention developers and evaluators to identify problems with implementation during the feasibility stage and to develop strategies to potentially improve fidelity and engagement.

#### Implications for PRIDE

The strategies outlined in this manuscript, together with the findings from this study can be used to improve training for providers of PRIDE. This would ensure that healthcare providers have the required skills, attributes and facilitative work environment to deliver and adapt PRIDE as planned.

For people with dementia and their supporters, the findings highlight difficulties people face when trying to engage in activities with PRIDE. These factors may also be relevant for people with dementia and supporters who did not take part in PRIDE, but who are trying to find ways to engage in activities. This knowledge can be used to ensure that interventions and services are developed and delivered in a way that reduces barriers and maximises people’s potential to engage.

#### Future research

This method could be replicated in other interventions to develop strategies to improve fidelity of delivery and engagement. Additionally, researchers could test whether the implementation of such strategies might improve fidelity of delivery and engagement. For example, the initial strategies to improve fidelity of delivery and engagement developed in this mixed methods study, could be iteratively revised following stakeholder feedback, implemented and evaluated in future versions of PRIDE.

## Conclusions

Fidelity of delivery and engagement are complex behaviours. This manuscript provides an example of how behavioural science principles and mixed-methods can be applied to complex interventions during the feasibility stage to measure, understand and potentially improve fidelity of delivery and engagement behaviours, thus potentially increasing the effectiveness of interventions and quality of life for participants.

## Supplementary information

**Additional file 1:**

**Additional file 2:**

## Data Availability

The datasets generated and/or analysed during the current study are not publicly available due to participant confidentiality but are available from the corresponding author on reasonable request.

## Abbreviations

BCW: Behaviour Change Wheel
COM-B model: Capability, Opportunity, Motivation – Behaviour model
MRC: Medical Research Council
PRIDE: Promoting Independence in Dementia
TDF: Theoretical Domains Framework
BCTs: Behaviour Change Techniques
BCTTv1: Behaviour Change Technique Taxonomy version 1
