# Supplementary material for: Developing strategies to improve fidelity of delivery of, and engagement with, a complex intervention to improve independence in dementia: a mixed methods study
Source: BMC Med Res Methodol. 2020 Jun 12;20:153. doi: 10.1186/s12874-020-01006-x (PMC7291463; doi:10.1186/s12874-020-01006-x)
Supplement: Supplementary file 1 — Additional file 1: [file 12874_2020_1006_MOESM1_ESM.docx]

**Supplementary file – Reporting standards**

**Good Reporting of a Mixed Methods Study (GRAMMS) checklist (O’Cathain et al, 2017).**

| Good reporting of a mixed methods study (GRAMMS) guidance | | Guidance met? (Section: Page number in this manuscript) |
| --- | --- | --- |
| 1 | Describe the justification for using a mixed methods approach to the research question | Yes (Introduction: p5) |
| 2 | Describe the design in terms of the purpose, priority and sequence of methods | Yes (Methods: p7) |
| 3 | Describe each method in terms of sampling, data collection and analysis | Yes (Methods: observational study/self-report study described from page 7-9 and qualitative study described from pages 9-12) |
| 4 | Describe where integration has occurred, how it has occurred and who has participated in it | Yes – to develop recommendation (Methods: described in part 3 of the manuscript – methods identified on page 12) |
| 5 | Describe any limitation of one method associated with the present of the other method | Yes (Discussion: p23) |
| 6 | Describe any insights gained from mixing or integrating methods | Yes – Results: developed recommendations on pages 16-17 (fidelity) and 20-21 (engagement). Also discussed in discussion section on pages 21-23. |

O’Cathain A, Murphy E, Nicholl J. The quality of mixed methods studies in health services research. *J Health Serv Res Policy.* 2008; 13:92-98.
