# Supplementary material for: Developing strategies to improve fidelity of delivery of, and engagement with, a complex intervention to improve independence in dementia: a mixed methods study
Source: BMC Med Res Methodol. 2020 Jun 12;20:153. doi: 10.1186/s12874-020-01006-x (PMC7291463; doi:10.1186/s12874-020-01006-x)
Supplement: Supplementary file 2 — Additional file 2: [file 12874_2020_1006_MOESM2_ESM.docx]

**Supplementary files**

Supplementary file 1. Interview schedules for providers, with relevant COM-B and TDF domains

| **Interview questions** | **Prompts** | **COM-B** | **TDF constructs** |
| --- | --- | --- | --- |
| 1. Please tell me a bit about yourself and any thoughts you have about the PRIDE intervention. | - What things have you been engaged in with participants? - How has this changed over time – by participants |  |  |
| 1. How have you found the experience of delivering the PRIDE intervention? | - How difficult or easy is it to deliver the intervention? - Why? | Psychological capability |  |
| 1. Can you describe the PRIDE programme to me? | - What should be delivered in:   - Session 1   - Session 2   - Session 3 | Psychological capability |  |
| 1. How feasible do you think it is to deliver the intervention as it is described in the manual? | - Is it achievable? - Why/why not? - What would make it more feasible to deliver as planned? - Time required – is it reasonable? | Reflective motivation; | Optimism |
| 1. How do you think the PRIDE programme impacts on participants’ lives? | - No impact - Positive impact - Negative impact - Why? | Reflective motivation | Beliefs about consequences |
| 1. How do you feel about your ability to deliver the PRIDE programme as planned? | - What would help you feel more confident about your ability to deliver PRIDE as planned? | Reflective motivation | Beliefs about capability |
| 1. How comfortable do you feel delivering the intervention as planned? | - In what way? - What makes you feel uncomfortable (if anything)? - How? - What would help you to overcome these feelings? - Would you want to continue delivering the programme? | Automatic motivation | Emotion |
| 1. What strategies do you use to deliver the PRIDE intervention? | - Use of manual - Your own guide - Training in delivery of intervention - Support from PRIDE team - Sticking to it loosely/strictly - Why did you choose that method? | Physical capability; Psychological capability; | Skills  Cognitive skills, memory, attention and decision making, knowledge |
| 1. Thinking about the resources available to you, what has helped you deliver the PRIDE programme as planned? | - E.g. Training, your work environment, competing tasks or time constraints, support from PRIDE team - How? | Physical opportunity | Environmental context and resources |
| 1. Thinking about the people around you, who has helped you to deliver the PRIDE programme as planned? | - E.g. Participants you work with, co-workers, trainers, line-managers, researchers - How? - Which have you found has been most useful in helping you to deliver PRIDE as planned? | Social opportunity | Social influences |
| 1. Has anything got in the way of you delivering the PRIDE programme as planned? | - The resources available to you: e.g. not enough training, your work environment, competing tasks or time constraints (patient needs/session timing) - How? - Did you overcome any of these challenges? - If so, how? | Physical opportunity  Social opportunity  Automatic motivation | Environmental context and resources  Social influences  Reinforcement |
| 1. What could be changed in future to help you deliver the PRIDE programme as planned? | - What would you change? - Why? |  |  |
| 1. How important is it for you to deliver the PRIDE programme as planned? | - Why is it important? - Professionally? - Personally? | Reflective motivation  Automatic motivation | Goals, social professional, role and identity, intentions,  Reinforcement |
| 1. Are there any systems that are in place for monitoring whether you have delivered the intervention as planned? | - E.g. recording the sessions/filling out and returning checklists? - How did you find these systems? (easy/difficult/stressful) - What would make it easier? | Psychological capability | Behavioural regulation |
| 1. Is there anything else that you would like to say about the issues we have talked about? |  |  |  |

Supplementary file 2. Interview schedule for people with dementia and supporters, with relevant COM-B and TDF domains

| **Interview questions** | **Prompts** | **COM-B** | **TDF domains** |
| --- | --- | --- | --- |
| 1. Please tell me a bit about yourself |  |  |  |
| 1. Please tell me your experience of taking part in the PRIDE programme | - Overall experience - Three sessions - E.g. enjoyable/not enjoyable/interesting - Why? - What does taking part mean to you? - Has taking part in the programme impacted on your life? - If so, how? | Reflective motivation | Beliefs about consequences, social, professional role and identity |
| 1. How many of the three sessions did you take part in? | **If all three**   - What did you get out of the sessions?   **If one or two**   - What did you get out of the sessions? - Why did you only take part in one or two sessions? - Could anything be changed to make you want to take part more?   **If none**   - Why did you choose not to attend the sessions? - Could anything be changed to make you want to take part more? |  |  |
| 1. What do you think of the information you were given: (a) in the manual, and (b) by your dementia advisor in the sessions? | - How easy or difficult was it to understand? - What was it that made it easy/difficult to understand? - Was it relevant to you? - How did the information help/get in the way of you doing your activity? - What would make it easier/more relevant/helpful for you? | Psychological capability, physical capability;  Reflective motivation; | Physical, cognitive skills and knowledge.  Beliefs about capabilities |
| 1. What activity did you choose to work on? | - Why did you choose that activity?   **If participant did not choose to work on an activity**  - Why did you decide not to choose an activity to work on?   - Could anything be changed to make you want to choose an activity to work on? | Motivation (automatic/reflective) |  |
| 1. What strategies have you used to put your plan into practice? | - E.g. reminders, use of technology, - Why did you choose that strategy? | Psychological capability | Memory, attention and decision processes |
| 1. How did it go when you tried to put your plan into practice? | - E.g. well, not very well - Why? - What helped?   - Knew how to do it, had the right resources, wanted to do it, incentives   - How did it help? - What got in the way?   - Didn’t know how, time, didn’t have the right resources - What would need to make it easier for you? | Physical capability. Psychological capability.  Physical opportunity  Automatic motivation    Reflective motivation | Environmental context and resources  Reinforcement, goals  Beliefs about capabilities |
| 1. What could be changed to help you to do more of the activities you planned? | - What would you change? - Why? |  |  |
| 1. What makes it worthwhile for you to do the activities that you enjoy? | - How? - What difference would it make to your life if you could do your activities? | Reflective/automatic motivation | Beliefs about consequences |
| 1. What did you like or not like about the activities you chose? | - Why? | Motivation (automatic/reflective) | Emotions |
| 1. Thinking about the people around you, who helped you to do the activities you planned? | - E.g. DAW, friends, family - How did they help? - How could your situation change to make it easier? - What kind of support would you need? | Social opportunity | Social influences |
| 1. What was your relationship with your DAW like? | - E.g. get on well/had some issues - What was the reason for that? - How could it be improved? | Social opportunity | Social influences |
| 1. How did you find filling in the forms for the programme? | - E.g. easy, difficult, stressful - PRIDE: your experience forms - Plan, do, review forms - What would make it easier? | Psychological capability | Behavioural regulation |
| 1. Is there anything else that you’d like to say about what we have talked about? |  |  |  |

*Supplementary file 3.* Fidelity of delivery scores for standardised PRIDE components across different sources of rating, sessions and sites

|  | **Mean % of components delivered (range)** | | | | | | | | |
| --- | --- | --- | --- | --- | --- | --- | --- | --- | --- |
|  | **Audio-recordings** | | | **Provider** | | | **Participant** | | |
| **Site** | **Session 1** | **Session 2** | **Session 3** | **Session 1** | **Session 2** | **Session 3** | **Session 1** | **Session 2** | **Session 3** |
| **Overall** | 69.2  (13.6-86.4) | 57.7  (41.7-83.3) | 54.9  (25-95.8) | 85.0  (22.7-100) | 84.3 (61.1-100) | 86.5  (62.5-100) | 89.8  (59.1-100) | 90.14  (50-100) | 92.5  (50-100) |
| A | 62.5  (13.6-84.1) | 56.1  (50-63.9) | 51.0  (33.3-66.7) | 92.5  (72.7-100) | 87.7  (80.6-100) | 75.6  (62.5-91.7) | 97.0  (90.9-100) | 85.6  (50-100) | 93.1  (79.2-100) |
| B | 75.8  (47.7-84.1) | 61.1  (44.4-86.1) | 63.9  (54.2-70.8) | 69.3  (22.7-90.9) | 72.2  (61.1-88.9) | 70.8  (70.8) | 89.8  (75-90.9 | 79.2  (58.3-100) | 87.5  (87.50) |
| C | 73.1  (70.5-79.6) | 58.8  (41.7-83.3) | 56.7  (25-95.8) | 86.2  (54.6-100) | 82.3  (66.7-94.4) | 93.5  (75-100) | 83.2  (59.1-100) | 88.9  (80.6-100) | 88.2  (50-100) |
| D | 60.2  (20.5-86.4) | 50  (44.4 (55.6) | 50.8  (25-75) | 84.7  (77.3-93.2) | 88.5  (77.8-100) | 93.8  (79.2-100) | 94.9  (90.91-97.7) | 98.9  (94.4-100) | 97.2  (91.7-100) |
| Unknown |  |  |  |  |  |  | 92.6  (70.5-100) | 100  (100) | 97.2  (91.7-100) |
| *^Note:^* ^N/A (Not applicable) scores were scored as not done (0)^  ^Max fidelity (100%): Session 1: n=44, Session 2: n=36, Session 3: n=24^  ^Blank cells indicate that either the sets were not sampled for rating (audio-recordings) or that data were missing.^  ^To ensure site anonymity, site numbers have been shuffled up so that sites 1-4 do not directly correspond to letters A-D^ | | | | | | | | | |

*Supplementary file 4*. Fidelity of delivery scores for tailored topics and components across different sources of rating, sessions and sites

| **Site** | **Mean number delivered (range)** | | | |
| --- | --- | --- | --- | --- |
|  | **Researcher** | | **Provider** | |
|  | **Session 1** | **Session 2** | **Session 1** | **Session 2** |
| **Overall**  Topics  Components |  |  |  |  |
|  | 1.9 (0-4) | 1.1 (0-2) | 2.4 (0-7) | 2.2 (0-7) |
|  | 4.6 (0-16) | 3.0 (0-8) | 7.9 (0-29) | 7.8 (0-45) |
| A  Topics  Components |  |  |  |  |
|  | 1.5 (0-3) | 0.8 (0-2) | 1.9 (1-3) | 1.9 (1-3) |
|  | 6.8 (0-16) | 2.8 (0-5) | 7.9 (3-18) | 5.1 (3-7) |
| B  Topics  Components |  |  |  |  |
|  | 1.8 (0-5) | 1.2 (0-3) | 0.8 (0-1) | 0 (0) |
|  | 2.8 (0-11) | 2.2 (0-7) | 4 (0-6) | 0 (0) |
| C  Topics  Components |  |  |  |  |
|  | 2.2 (1-4) | 1.5 (0-2) | 1.5 (0-4) | 2 (0-5) |
|  | 5.7 (4-7) | 4.8 (0-8) | 5.4 (0-14) | 6.5 (0-12) |
| D  Topics  Components |  |  |  |  |
|  | 1.8 (0-3) | 0 (0) | 5.4 (3-7) | 3.7 (1-7) |
|  | 3.3 (0-8) | 0 (0) | 14.1 (5-29) | 15.3 (5-45) |
| ^Note:^  ^Blank cells indicate that either the sets were not sampled for rating (audio-recordings) or that data were missing.^  ^To ensure site anonymity, site numbers have been shuffled up so that sites 1-4 do not directly correspond to letters A-D^ | | | | |

Supplementary file 5. Participants’ engagement (receipt and enactment) with the PRIDE intervention across the three sessions and sites

|  |  | **Mean % (range)** | |
| --- | --- | --- | --- |
| **Session** | **Site** | **Receipt** | **Enactment** |
| 1 | Overall | 85.9 (0-100) | - |
|  | A | 83.3 (50-100) | - |
|  | B | 87.5 (50-100) | - |
|  | C | 75 (0-100) | - |
|  | D | 100 (100) | - |
|  | Unknown | 93.8 (75-100) | - |
| 2 | Overall | 87.5 (50-100) | 81.3 (0-100) |
|  | A | 90 (50-100) | 80 (0-100) |
|  | B | 75 (50-100) | 75 (75) |
|  | C | 85.7 (75-100) | 92.9 (75-100) |
|  | D | 90 (50-100) | 65 (50-100) |
|  | Unknown | 100 (100) | 100 (100) |
| 3 | Overall | 90.6 (50-100) | 82.8 (0-100) |
|  | A | 91.7 (75-100) | 66.7 (0-100) |
|  | B | 50 (50) | 75 (75) |
|  | C | 87.5 (50-100) | 83.3 (50-100) |
|  | D | 100 (100) | 100 (100) |
|  | Unknown | 100 (100) | 83.3 (75-100) |
| *^Note^*^: Responses that were unclear were coded as missing and thus scored as ‘0’ in percentage calculations.^  ^-: Indicates that there were no scores for enactment for Session one as enactment was only measured in Sessions two and three.^ | | | |

Supplementary file 6. Frequency of occurrences of COM-B domains across themes and sub-themes for fidelity of delivery

| **Theme** | **Sub-theme** | **Psychological Capability** | **Physical Capability** | **Physical opportunity** | **Social opportunity** | **Automatic motivation** | **Reflective motivation** |
| --- | --- | --- | --- | --- | --- | --- | --- |
| 1. Providers’ knowledge | Overall | n=101 (B: 59, F: 65) | n=0 | n=93 (B: 65, F: 49) | n=65 (B: 40, F: 46) | n=32 (B: 23, F: 13) | n=35 (B: 9, F: 33) |
|  | 1) Prior knowledge | n=32 (B: 11, F: 27) | n=0 | n=17 (B: 10, F: 12) | n=25 (B: 18, F: 19) | n=11 (B: 5, F: 8) | n=9 (B: 3, F: 9) |
|  | 2) Skills to deliver | n=66 (B: 43, F: 40) | n=0 | n=67 (B: 46, F: 35) | n=42 (B: 25, F: 29) | n=19 (B: 13, F: 13) | n=23 (B: 5, F: 22) |
| 1. Providers’ attributes | Overall | n=70 (B: 43, F: 41) | n=0 | n=73 (B: 48, F: 44) | n=90 (B: 48, F: 65) | n=68 (B: 35, F: 41) | n=84 (B: 18, F: 78) |
|  | 3) Beliefs about PRIDE as part of job | n=22 (B: 14, F: 11) | n=0 | n=18 (B: 8, F: 14) | n=31 (B: 20, F: 15) | n=11 (B: 6, F: 7) | n=15 (B: 4, F: 12) |
|  | 4) Personal characteristics | n=19 (B: 10, F 13) | n=0 | n=28 (B: 15, F: 17 | n=42 (B: 21, F: 35 | n=30 (B: 9, F: 24) | n=72 (B: 12, F: 69) |
|  | 5) Feelings about delivery | n=51 (B: 33, F: 28) | n=0 | n=48 (B: 35, F: 28) | n=40 (B: 22, F: 27) | n=56 (B: 30, F: 32) | n=37 (B: 13, F: 32) |
| 1. Adaptation of PRIDE in relation to participants’ needs | Overall | n=53 (B: 27, F: 35) | n=1 (B: 1) | n=66 (B: 39, F: 42) | n=118 (B: 86, F: 95) | n=21 (B: 11, F: 13) | n=53 (B: 20, F: 44) |
|  | 6) Ease of adaptation with fidelity | n=43 (B: 21, F: 30) | n=0 | n=56 (B: 30, F: 38) | n=81 (B: 58, F: 68) | n=16 (B: 9, F: 10) | n=44 (B: 17, F: 37) |
|  | 7) Participant engagement | n=24 (B: 15, F: 13) | n=1 (B: 1) | n=20 (B: 13, F: 11) | n=70 (B: 56, F: 57) | n=7 (B: 4, F: 4) | n=22 (B: 8, F: 18) |
| 1. Logistical considerations | Overall | n=46 (B: 33, F: 27) | n=0 | n=98 (B: 63, F: 61) | n=118 (B: 65, F: 97) | n=25 (B: 12, F: 15) | n=34 (B: 8, F: 30) |
|  | 8) Organisational constraints | n=16, (B: 11, F: 12) | n=0 | n=42 (B: 28, F: 29) | n=26 (B: 17, F: 17) | n=8 (B: 3, F: 7) | n=7 (B: 2, F: 6) |
|  | 9) Social support for delivery | n=20 (B: 14, F: 10) | n=0 | n=27 (B: 16, F: 19) | n=85 (B: 39, F: 75) | n=12 (B: 5, F: 8) | n=20 (B: 5, F: 18) |
| ^Note: Frequency: n= overall frequency, B: barrier F: facilitator^ | | | | | | | |

Supplementary file 7: COM-B and thematic analysis examples for fidelity of delivery

| **Theme** | **Subtheme** | **An example of barrier/facilitator** | **Psychological Capability** | **Physical Capability** | **Physical opportunity** | **Social opportunity** | **Automatic motivation** | **Reflective motivation** |
| --- | --- | --- | --- | --- | --- | --- | --- | --- |
| 1. Providers’ knowledge | 1) Prior knowledge | Barrier | E.g. Lack of knowledge on what to do from current role and about the participant | N/A | E.g. Lack of use of strategies used in role (e.g. note taking), not having time to meet participants prior to delivery | E.g. need to familiarise due to swapping between PRIDE and job role, not having met participant prior to delivery | E.g. Worries about knowing how to deliver/doing it right as it is a new part of role | E.g. evaluation that experience influences knowledge, belief to trust in prior knowledge to know how to deliver |
|  |  | Facilitator | E.g. Prior knowledge of participant, working with people with dementia, and resources/ environment | N/A | E.g. manual as useful icebreaker with no prior knowledge of participant, prior work strategies helpful for delivery | E.g. participant engagement, met participant prior to delivery | E.g. Experience as increasing confidence | E.g. Evaluation that experience influences knowledge for delivery, belief to trust in own prior knowledge to know how to deliver |
|  | 2) Skills to deliver | Barrier | E.g. Lack of knowledge on how to deliver the intervention as specified in manual, Lack of memory of information from training | N/A | E.g. lack of time in training session to practice/learn everything, not knowing how to use manual or Dictaphones | E.g. Involvement of supporter, knowledge of participant, lack of guidance from PRIDE researchers, lack of knowledge on tailoring | E.g. Anxiety about manual/not knowing how to deliver and time since training/audio-recordings | E.g. Negative beliefs about delivering something for first time |
|  |  | Facilitator | E.g. Increased experience and skills to fill in forms | N/A | E.g. Resources (e.g. instruction sheet/own prompts), opportunity to practice, training day | E.g. resources/support from researchers, participant engagement, Researchers giving sheet on how to deliver PRIDE, support from other DAWs | E.g. Delivering PRIDE enjoyable and comfortable once know what doing | E.g. Understanding about what PRIDE is and why it’s important |
| 1. Providers’ attributes | 3) Beliefs about PRIDE as part of job | Barrier | - E.g. Not remembering how to deliver until thought of in relation to job, not knowing how to deliver/difficult to deliver (outside of remit), difficult not to blur job role and PRIDE together | N/A | E.g. not familiar with manual/not knowing how to use it, training as not providing skills in the same way as job, possibility of sessions being longer due to work related issues | E.g. work remit changing, needing to deal with other job related issues whilst delivering PRIDE, needing shared knowledge from other DAWs for delivery | E.g. Delivery as daunting until realise it’s something they already do, not as natural as delivering job role | E.g. Belief that PRIDE not right for DAW |
|  |  | Facilitator | - E.g. thinking of it as more formal version of job helped to remember how to deliver, experience delivering PRIDE facilitating knowledge, knowledge of how to deal with difficulties from job as useful, relevant knowledge | N/A | E.g. Not having lots of paperwork unlike job, not familiar with manual/knowing how to use it, easier if delivered as part of job | E.g. Needing other people to help know how to delivery, easier to deliver if part of job, experience from job as helpful, maintaining separation (different DAW deals with participant outside of intervention) | E.g. Exciting to get involved in new evidence based interventions, enjoy seeing people with dementia and delivery | E.g. Belief that it’s good to get involved in new things and that there is value in PRIDE above and beyond role |
|  | 4) Personal characteristics | Barrier | E.g. Individual differences in knowledge, learning style and anxieties about doing it right | N/A | E.g. approach to using manual, views on manual and training, level of instruction | E.g. type of provider and participant influence use of manual, personal styles relating to delivery | E.g. Feelings towards delivering PRIDE as differing | E.g. Beliefs about the use of PRIDE, and delivery |
|  |  | Facilitator | E.g. Differences in learning style for training and experience | N/A | E.g. Approach to delivery as influenced by checklist (because of personality), use of personal strategies | E.g. Personal styles to delivery in relation to tailoring the intervention to participants and to delivery | E.g. wanting to deliver everything, enjoying spending time with people with dementia | E.g. Beliefs about the use of PRIDE as useful for people living with dementia (personally and professionally) |
|  | 5) Feelings about delivery | Barrier | E.g. Anxiety about not knowing how to deliver PRIDE/whether doing it right, using Dictaphones and delivering the first session | N/A | E.g. Negative feelings in relation to manual and audio-recorders, time since training increasing nerves, tiring fitting, tiring trying to fit delivery in around full time job | E.g. feeling as though not coming across well to participants, worries about participant expectations, and tailoring, and delivering when do not know participant | E.g. anxiety about delivery, audio-recordings and delivering certain topics. Manual as off-putting | E.g. Belief about needing to deliver it as planned, Evaluating delivering PRIDE as planned as restricted |
|  |  | Facilitator | E.g. Delivery as going better than expected – manual becoming friend is easier to deliver, familiarity with delivery increased confidence | N/A | E.g. needing time to familiarise, experience, sticking to manual as comfortable / being more flexible over time with experience (increased confidence) | E.g. support from other DAWs reducing anxieties, encouragement from researchers helpful, worries relating to delivering without supporter | E.g. enjoying delivery and feeling more comfortable sticking to the manual | E.g. Belief that PRIDE is useful and plans to deliver as planned |
| 1. Adaptation of PRIDE in relation to participants’ needs | 6) Ease of adaptation with fidelity | Barrier | E.g. set structure and delivery of some topics and aspects of the manual as difficult, lack of knowledge | N/A | E.g. time needed to get used to manual which becomes easier to deliver, manual restricting | E.g. Changing approach to use of manual and content of intervention depending on participant | E.g. worries about how participants view providers, and upsetting participants | E.g. planning to deliver it however best for participants, evaluations that delivering with fidelity is unrealistic in dementia interventions |
|  |  | Facilitator | E.g. set choices as helpful for knowledge, experience, ability, some topics easier to deliver | N/A | E.g. Resources (session guide/manual) useful for delivery | E.g. changing approach to delivery and content of intervention depending on participant engagement | E.g. more comfortable with less fidelity or sticking to delivering manual as outlined | E.g. Belief that delivery depends on type of dementia, evaluations on benefits of delivery, plan to deliver it however best for participant |
|  | 7) Participant engagement | Barrier | E.g. Some aspects of PRIDE as confusing to deliver, lack of participant engagement | E.g. Sick leave | E.g. manual as a resource to use in relation to participants, time needed (differs depending on engagement) | E.g. Participant engagement/wants as influencing delivery | E.g. Not wanting to upset participants with less awareness/engagement | E.g. Belief that lack of supporter meant that there was no clear definition, evaluation that delivering with fidelity meant sessions flowed less/less engagement |
|  |  | Facilitator | E.g. Participant engagement = easier to deliver, some topics easier to deliver than others | N/A | E.g. Not needing to cancel any sessions, manual facilitating choices/engagement, changing use of manual with different participants, time taken to deliver differed for participants | E.g. Participant engagement/wants as influencing delivery | E.g. feeling rewarded from delivery, happy that participants engaged | E.g. Evaluation that PRIDE can still work without awareness into dementia, beliefs about PRIDEs usefulness |
| 1. Logistical considerations | 8) Organisational constraints | Barrier | E.g. too much information to deliver (1^st^ session), uncertainty of delivering, difficult to remember PRIDE and jobs – too much time in between | N/A | E.g. difficulties making appointments due to busy calendars, too much time in between, participants need more sessions, difficult to find time to deliver PRIDE alongside full time job | E.g. Delivery as more within another service remit, needing to support both people – lots of other topics coming up (makes time longer), timing variability due to participant/DAW availability | E.g. Anxieties as making delivery longer, working full time and delivering PRIDE as draining | E.g. Belief that PRIDE needs to be more tightly structured |
|  |  | Facilitator | E.g. Familiarity with PRIDE, strategies for delivery | N/A | E.g. arranging own calendars, fitting well around work commitments, nothing competing for time, more time to deliver when working part time | E.g. Facilitative work environment – able to manage own diaries | E.g. Time needed to feel familiar and confident, liking delivering PRIDE(conflict with time availability) | E.g. Belief that no paperwork is a good thing and that delivery would be smoother if PRIDE took on by organisations |
|  | 9) Social support for delivery | Barrier | E.g. needing peer support, difficult to deliver without others input, | N/A | E.g. Resource format not suitable for providing support for delivery, not enough support with Dictaphones, training too long before actual delivery | E.g. needing more support with technical processes, involvement of support, lack of participant engagement as barrier | E.g. delivery as unnatural, needing reassurance, Dictaphones not working as upsetting | E.g. Lack of supporter for delivery – belief that harder to deliver |
|  |  | Facilitator | E.g. need specific guidance on how to deliver, difficult to support both people in the time allowance, peer support/researcher support as helpful for knowledge | N/A | E.g. resource provision by researchers, support from DAWs to go through paperwork | E.g. Researchers as helpful – provision of materials/support, DAW support– reducing anxieties, Participant/supporter engagement | E.g. enjoying seeing people with dementia, and delivering, surprised that it works | E.g. Belief that shared experience was helpful |

Supplementary file 8. Frequency of occurrences of COM-B domains across themes and sub-themes for engagement

| **Theme** | **Sub-theme** | **Psychological capability** | **Physical capability** | **Physical opportunity** | **Social opportunity** | **Automatic motivation** | **Reflective motivation** |
| --- | --- | --- | --- | --- | --- | --- | --- |
| I. Participants’ attributes | Overall | N=55 (B:48, F: 11) | N=22 (B:15, F: 10) | N=83 (B: 48, F: 50) | N=96 (B: 17, F: 89) | N=108 (B: 44, F: 82) | N=127 (B: 30, F: 108) |
|  | 1. Preferences for PRIDE activities | n=29 (B: 25, F: 8) | n=17 (B: 11, F: 8) | n=47 (B: 27, F: 27) | n=43 (B: 13, F: 37) | n=72 (B: 27, F: 60) | n=46 (B: 8, F: 42) |
|  | 1. Beliefs about PRIDE | n=20 (B: 17, F: 4) | n=1 (B: 1) | n=29 (B: 15, F: 18) | n=38 (B: 5, F: 37) | n=16 (B: 4, F: 13) | n=67 (B: 18, F: 56) |
|  | 1. Feelings about PRIDE | n=20 (B: 20, F: 2) | n=10 (B: 6, F: 6) | n=29 (B: 17, F: 20) | n=42 (B: 5, F: 39) | n=52 (B: 27, F: 35) | n=35 (B: 7, F: 31) |
| II. Participants’ capability | Overall | n=73 (B: 68, F: 12) | n=22 (B: 16, F: 9) | n=53 (B: 30, F: 34) | n=41 (B: 11, F: 37) | n=43 (B: 22, F: 29) | n=36 (B: 11, F: 28) |
|  | 1. Physical health | N=3 (B: 3, F: 1) | N=16 (B: 14, F: 5) | N=9 (B: 8, F: 3) | N=6 (B: 2, F: 6) | N=9 (B: 4, F: 7) | N=4 (B: 0, F: 4) |
|  | 1. Cognitive factors | N=72 (B: 67, F: 12) | N=8 (B: 4, F: 5) | N=48 (B: 26, F: 32) | N=37 (B: 9, F=33) | N=37 (B: 19, F: 25) | N=33 (B: 11, F: 25) |
| III. Opportunity to engage | Overall | n=81 (B: 66, F: 24) | n=25 (B: 20, F: 10) | n=159 (B: 94, F: 99) | n=172 (B: 35, F: 156) | n=70 (B: 34, F: 47) | n=86 (B: 15, F: 75) |
|  | 1. Accessibility | n=57 (B: 47, F: 16) | n= 13 (B: 9, F: 7), | n=130 (B: 78, F: 78) | n=53 (B: 15, F: 47) | n=33 (B: 18, F: 19) | n=44 (B: 11, F: 36) |
|  | 1. Support | n=44 (B: 36, F: 14) | n=15 (B: 12, F: 6) | n=56 (B: 26, F: 40) | n=155 (B: 30, F: 142) | n=42 (B: 19, F: 30) | n=45 (B: 8, F: 40) |
|  | 1. Activity characteristics | n=14 (B: 10, F: 6) | n=5 (B: 4, F: 2) | n=21 (B: 13, F: 14) | n=22 (B: 5, F: 20) | n=16 (B: 8, F: 13) | n=10 (B: 2, F: 9) |
| Frequency: n= overall frequency, B: barrier, F: facilitator | | | | | | | |

Supplementary file 9: COM-B and thematic analysis examples for engagement

| **Theme** | **Subtheme** | **Barrier/ facilitator** | **Psychological Capability** | **Physical Capability** | **Physical opportunity** | **Social opportunity** | **Automatic motivation** | **Reflective motivation** |
| --- | --- | --- | --- | --- | --- | --- | --- | --- |
| I. Participants’ attributes | 1. Preferences for PRIDE activities | Barrier | E.g. difficulty and memory stopping doing activities liked | E.g. physical health stopping doing activities liked | E.g. lack of resources, appropriate location, time and money to do activities liked | E.g. unable to do activity without support, other people distracting from activity | E.g. feelings of embarrassment, lack of enjoyment or want to do activity. Not liking certain types of transport, or worrying about resources breaking | E.g. evaluations of intervention (supporter not knowing what’s going on and cannot support) or activities (location not appropriate) |
|  |  | Facilitator | E.g. ease of taking part in PRIDE and doing activities | E.g. having the physical ability to do liked activities | E.g. location appropriate to do liked activities, manual (resource) helping to locate activities, weather facilitating | E.g. Support to help organise activity, having people to do activity with | E.g. As enjoying/liking and wanting to do activities | E.g. being interested in activities, activities needing to have an end, beliefs about activities being good |
|  | 1. Beliefs about PRIDE | Barrier | E.g. little knowledge of what intervention is, confusion between PRIDE and other support services | E.g. Inability of participant to drive anymore putting pressure on supporter to drive to support daily activities | E.g. physical location as not appropriate, needing a summary document between sessions, PRIDE taking time away from other activities and PRIDE as not providing information | E.g. Supporter as not unable to take part as participant wanted to do it on own | E.g. Missing doing activities used to do (PRIDE gives new ideas) | E.g. belief that intervention not as helpful for supporter if not involved, belief that PRIDE may not be understandable to everyone, belief that PRIDE was not providing enough information |
|  |  | Facilitator | E.g. Beliefs that manual is helpful as participant is only just becoming familiar with dementia diagnosis and belief that PRIDE is easy to understand. | N/A | E.g. PRIDE resources helpful for finding activities and easy to follow, location easy, summary document would be helpful | E.g. Having someone to talk to as helpful, providing encouragement needed | E.g. Liking activities in manual and liking PRIDE | E.g. beliefs that PRIDE is relevant, interest in activities, evaluation of benefits of PRIDE |
|  | 1. Feelings about PRIDE | Barrier | E.g. Worries relating to not remembering the intervention/demented diagnosis  Worries about not remembering the intervention/dementia diagnosis. Not being able to do activities liked due to diagnosis and memory | E.g. physical health making it difficult to do activities that would like to do, health appointments preventing from doing activities | E.g. liking/wanting to do activities but having no way of getting there/not wanting to go too far, worries about driving, daunting PRIDE resources, | E.g. lack of support for getting to activities, not liking people at activity, frightened to do activity on own | E.g. feelings of negativity talking to people, anxiety to do activities, manual as exhausting | E.g. Prior evaluations of what the activity may have been like |
|  |  | Facilitator | E.g. feeling pleased with activity as easy to do | E.g. liking to do activities that physically able to do | E.g. wanting to find an easily accessible bridge club, having an accessible place to do liked activity, activities feeling like a safe environment | E.g. DAW helping to overcome worries, supporter helping to do activities like to do | E.g. liking to do activities and liking PRIDE DAWs/researchers | E.g. evaluation of type of activities liked and positive evaluations of the PRIDE intervention |
| II. Participants’ capability | 1. Physical health | Barrier | E.g. Dementia limiting ability to do physical activity, or other conditions as limiting concentration | E.g. physical health problems/appointments limiting ability to do activities | E.g. lack of time due to health appointments, activities taking longer | E.g. needing other people to help due to physical health | E.g. anxiety of doing activity on own, not liking driving, not wanting to have a walking aid | N/A |
|  |  | Facilitator | E.g. able to do activity easily despite concentration (social support) | E.g. Physical health as enabling to do activities | E.g. weather, ease of use of resource | E.g. doing activities with someone else | E.g. Pleased with activity, like doing activities that keep going health wise | E.g. positive evaluation of activities, plans to do activities to keep physically healthy |
|  | 5) Cognitive factors | Barrier | E.g. Memory of PRIDE / DAW / diagnosis, reading ability, finding words to communicate, knowledge of how to do activity / use technology / diagnosis, difficulties understanding PRIDE | E.g. Unable to walk to activities and difficulties using public transport, difficulties doing activities alone due to physical health | E.g. time, location, complexity of resources, lack of summary document, other competing activities | E.g. having no one to do activities with, not liking people at activity, not knowing who is who (PRIDE vs other support) | E.g. lack of confidence in communication, not enjoying activities first time, embarrassment if unable to do activity. Manual as exhausting to read | E.g. Evaluation that denied diagnosis until PRIDE, as not planning but just doing, evaluation of not being able to do activities that are too difficult |
|  |  | Facilitator | E.g. ability to understand manual, and knowing how to do activities once done it once, activity is easy to do easy to do | E.g. Ability to do physical activities still know how to do | E.g. PRIDE resources helpful for memory, manual easy to understand | E.g. DAW provision of PRIDE resources to help memory, asking for help if not know how to do it, availability of other people to do activity with | E.g. liking to do activities/liking activities but not knowing what to do | E.g. Positive evaluations of PRIDE giving ideas, belief that doing activity will help memory |
| III. Opportunity to engage | 6) Accessibility | Barrier | E.g. difficulties organising activities due to memory, lack of knowledge on how to use public transport, or to find places, needing a summary to know what activities chosen, forgetting to write down activities | E.g. Physical health as making it difficult to get anywhere to do activities | E.g. Weather, time, location/transport, and expense of activities as barriers to activities, lack of PRIDE summary, length of manual and paperwork is tedious | E.g. People not providing support to get somewhere and having no one to do activity with | E.g. anxieties of travelling, manual as exhausting | E.g. Evaluation that some available places aren’t appropriate/others aren’t accessible, belief that manual as difficult to understand for some people, evaluation that PRIDE paperwork is tedious, evaluation that PRIDE is more questions than information |
|  |  | Facilitator | E.g. ability to understand information, get to places once been there before and activities and resources easy to do / use | E.g. Physical health as helping to get to activities | E.g. Accessible/appropriate location, prompts (reminders/diary/to do sheets) and weather conditions | E.g. DAW/supporter helping to organise activity and getting to activity, and having someone to do activity with | E.g. Liking to skim through manual, wanting to do activities but not knowing how to get there/having somewhere accessible, liking activity | E.g. Evaluation that PRIDE is relevant and useful |
|  | 7) Support | Barrier | E.g. dementia requiring participants to move closer to family, not knowing what to do and needing help, not knowing who is PRIDE and who is other support, not knowing what participants need support with due to lack of involvement | E.g. Needing support to help people to get to places by transport | E.g. needing support to find places to do activity, needing support to find out about resources | E.g. not having anyone to do activity with, not being able to do activity on own | E.g. not wanting to talk to people who are not close friends in case of embarrassment, e.g. anxiety to do activity prior to support | E.g. evaluation that PRIDE was more helpful for participant but not enough support from DAW for supports not involved – need summary |
|  |  | Facilitator | E.g. Support helping to know how to do activities | E.g. Support as helping to get to places / do activities | E.g. Support to organise activities and find places to do activities, manual for prompt to choosing activities (do not need support) | E.g. Support from DAW/supporter/neighbour/organisations as helpful to do activities (emotional/practical) | E.g. wanting some support on to do activities, looking forward to seeing DAW, looking forward to activity – good support network | E.g. Evaluation that speaking to DAW /family member was helpful |
|  | 8) Activity characteristics | Barrier | E.g. Difficulty of activities, length of activities and difficulty filling in calendar every day | E.g. activities too difficult for health, take longer due to physical health | E.g. lack of accessibility of activity, frequency of activity, length of activity | E.g. Not having people to do activity with as barrier | E.g. not liking things which don’t have an end | e.g. Evaluation that some activities with characteristics are harder to do |
|  |  | Facilitator | E.g. ease of activities | E.g. physical health facilitates some activities done previously | E.g. Accessibility of activity, frequency of activity, length of activity | E.g. Having people to do activity with as facilitator, support helping simplify the activity | E.g. liking things which have an end/to win | E.g. evaluation of types of activities liked (with an end/can do) |
|  | | | | | | | | |
